# Flooding in Nigeria, towards prioritizing mental health and psychosocial support

**DOI:** 10.11604/pamj.2022.43.199.38251

**Published:** 2022-12-22

**Authors:** Ejike Malachy Oluka, S. Benedict Dossen, Ikenna Desmond Ebuenyi

**Affiliations:** 1 Department of Clinical Skills, St. George´s University, True Blue, Grenada,; 2 Department of Mental Health, The Carter Center, Monrovia, Liberia,; 3 Department of Rehabilitation Science and Technology, University of Pittsburgh, Pennsylvania, USA

**Keywords:** Flooding, mental health, psychosocial support, rehabilitation, Nigeria

## Abstract

In the past decade, Nigeria has been experiencing worsening flooding. Beyond the physical injuries caused, it can impact the mental health of affected individuals. While new mental health disorders can emerge, exacerbation of preexisting mental conditions are common in the aftermath of flooding. Therefore, it is critical to integrate mental health and psychosocial support as part of the emergency response available to affected populations on both short-term and long-term basis.

## Opinion

According to the United Nations Children's Fund (UNICEF), about 34 out of the 36 States in Nigeria are currently experiencing flooding, making it the worst flooding Nigeria has seen in a decade [1]. Presently, over 600 persons have lost their lives and more than 1.4 million displaced from their homes [1]. As the heavy rains continue, more people are expected to be in dire need of humanitarian assistance to mitigate some of the negative effects of the flooding. As the most frequent type of natural disaster, flooding can be devastating as it leads to loss of life and destruction of property [2]. According to Nigeria National Emergency Management Agency (NEMA), the flooding in 2012 killed about 360 people while 2.3 million persons were displaced and the economic loss was in the amount of 2.5 trillion naira [3]. Another deadly consequence of flooding is the health impact on affected communities and individuals. Flooding is associated with numerous infectious diseases due to contamination of water sources which may lead to outbreaks of water-borne illnesses like cholera, dysentery, typhoid fever. So far, about 7,485 cases of cholera have been reported in three north-eastern States of Nigeria, with 319 associated deaths [1].

Flooding can either aggravate or trigger psychosocial distress due to associated socioeconomic losses experienced by affected individuals. Flooding can pose a significant psychological challenge to certain populations like children, elderly people, persons with disabilities and people with mental health conditions. Studies show they are prone to mental health disorders following stressful events like flooding [4,5]. Affected individuals have been noted to manifest different mental disorders like Post Traumatic Stress Disorder (PTSD), depression, anxiety, and in extreme cases, die by suicide [5]. The individual response to flooding is associated with feelings of grief and loss, which can be overwhelming and with various consequences. Increase in the abuse of substances and psychotropic medications use has been reported following flooding events. A study found an increase in the use of alcohol and tobacco following the 2011 Queensland flood in Australia [6]. Due to the pervasive nature of mental health problems, affected persons can continue to experience these symptoms years after the flooding; thus, the need to prioritize prompt recognition and response to the emotional burdens of flooding on the affected individuals.

Humanitarian response to the impact of flooding is often supported by national and international teams to support the various health needs of affected individuals. However, studies indicate that mental health and psychosocial support is often not prioritized [7]; and this may be worse in countries like Nigeria with high unmet need for mental health services. The final report of the International Federation of Red Cross and Red Crescent Societies (IFRC) on the September 2019 flooding in Nigeria showed the primary focus was on disbursement of cash and relief materials to the affected individuals and communities without any specialized mental health services offered [8]. While there is a national disaster response plan which delegates the provision of mental health services during emergencies to the Ministry of Health and NEMA [9]; however, evidence suggests that there is no Mental health and Psychosocial Support (MHPSS) implementation plan or framework in the national policy [10]. This implies that there may be missed opportunities to deliver MHPSS or uncoordinated delivery of MHPSS in humanitarian emergencies in the country [10]. The 1991 National mental health policy adopted by the Federal Ministry of Health (FMoH) had limited impact on account of implementation challenges [11], and there was no mention of MHPSS in it [10]. Also, a study found that the National mental health Action Committee (NMHAC) under the FMoH has no disaster preparedness plan for emergencies [10]. Similarly, the 2013 National Policy for Mental Health Service Delivery has no recommendations on delivery of MHPSS in humanitarian emergencies [12]. These gaps in policy recommendations implies that integration of MHPSS in disaster settings and the index flooding may not be prioritized even though research evidence supports it [2-5]. A report by the International Organization for Migration highlighted that MHPSS in Northeast Nigeria is very limited [13] and according to Adesina *et al*, although Nigeria has an MHPSS sub-working group (SWG), provision of services in humanitarian emergencies is limited [14]. Considering the high unmet need for human resources for mental health services in the country, affected individuals may receive no treatment or resort to traditional and religious leaders, who are untrained to deal with these challenges [10].

Therefore, we believe there is need to prioritize MHPSS for individuals affected by the present flooding and other humanitarian emergencies in the country; and we proffer the following recommendations.

### Recommendations

The flooding in Nigeria has elicited several responses from diverse stakeholders. However, it is essential that both governmental and non-governmental agencies work together to formulate an integrated approach targeted at vulnerable communities to address their mental health and psychosocial needs. To minimize the psychological impact of the floods, the following approaches are recommended as part of the overall flood response.

**Provision of daily basic needs:** following the flooding, most individuals affected are displaced from their homes and lose their means of livelihood. Early provision of food, water, shelter, security, and basic health care form the bedrock of their recovery, as lack of these can serve as secondary stressors to the individuals´ mental wellbeing [2]. Partners, International non-governmental organizations (INGOs), and Donors need to revisit their financial approval processes and make contingencies to release funds to address the needs of victims quickly. These can help reduce the emotional stress felt by the victims and reduce the mental health consequences. Also, the government needs to leverage support from both international and local partners and coordinate their response support better through the incident management team. Lessons learned from the COVID-19 pandemic can be used to ensure efficiency and effectiveness and to reduce waste.

**Figure 1:**
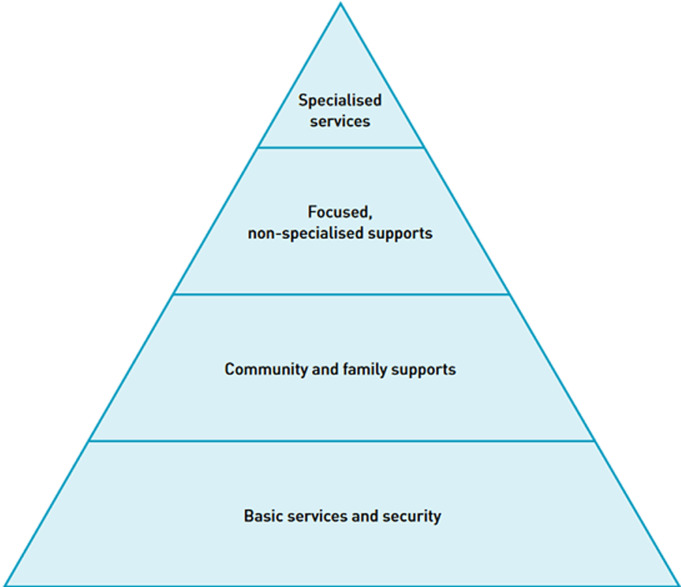
inter-agency standing committee Intervention pyramid for mental health and psychosocial support in emergencies

**Implementation of the Inter-agency standing committee (IASC)´s Mental Health and Psychosocial Support (MHPSS) guidelines:** the IASC-MHPSS guideline is a pyramid of interventions (Figure 1), which tailors the care provided to each individual´s mental health needs [15]. The relief organizations and agencies responding to the flood should adopt and implement the IASC-MHPSS guidelines for emergencies. This will ensure that affected victims of the floods receive appropriate care for their mental health needs at this time of heightened stress. As a component of the IASC-MHPSS guideline, it is crucial to provide the WHO Psychological First Aid (PFA) to the victims in the aftermath of the floods. Community Health Workers or members of the emergency response team can be rapidly trained to apply PFA for people in need. This will allow them to promptly identify people in need (LOOK), understand their issues better (LISTEN), and connect them to the services they need (LINK). This approach was shown to be effective in scaling up the number of mental health providers in Afghanistan between the period of 2002 to 2008 and more people received psychosocial support for violence related issues [10]. According to Tol *et al*. providing MHPSS in humanitarian settings is crucial for wellbeing [16] and we strongly recommend its inclusion in the mental health policy in Nigeria and allocation of funding for its implementation. This is relevant because according to the WHO, although 89% of countries reported MHPSS as part of their COVID-19 pandemic response, only 17% ensured funding for these activities [17]. Figure 1 [15].

**Training of mental health providers**: as there are only about 250 psychiatrists for a population of over 200 million in Nigeria and deficiency in community mental health services [18], it is imperative that provisions are made to train more professionals in this field to assist the already strained healthcare system to effectively respond to this humanitarian crisis. Establishment of community mental health centers will facilitate long term follow up of these vulnerable individuals and allow for early identification of mental illnesses triggered by the flood which may not be immediately evident [7]. The dearth of specialist mental healthcare providers implies that it is essential to adopt the use of non-specialist healthcare providers in Nigeria. The Mental Health Gap Action Programme (mhGAP) Humanitarian Intervention Guide is a simple and effective tool designed to provide first-line management for mental health and psychosocial support where specialist care is limited [19]. Following the 2015 earthquake in Nepal, some health professionals were trained to treat common mental health disorders with the mhGAP humanitarian intervention guide which was found to be successful in improving access to mental health services for victims of the emergency [20].

**Post flood rehabilitation of individuals and communities:** studies indicate that in the aftermath of flooding, affected individuals require rehabilitation to meet their basic needs such as housing, feeding, social, education and work and or employment [21,22]. The prioritization of rehabilitation is critical to recovery of affected individuals and prevention of the mental health consequences of flooding.

**Funding:** IDE was funded by University of Pittsburgh.

## Conclusion

Historical evidence and research support the integration of mental health services alongside health services in humanitarian settings especially during flooding disasters. Mental health services and support promotes emotional resilience of flood victims on their road to recovery and as such, makes it a vital component of every disaster response protocol. Hence, incorporation of MHPSS guidelines alongside the provision of basic physical needs is essential and urgent. Although, Nigeria has unmet need for mental health providers [18], efforts must be made to adopt evidence-based methods through the training and use of non-specialist health-care providers to support the heightened mental health needs created by flooding.
